# Analytical Solutions of Fractional-Order Heat and Wave Equations by the Natural Transform Decomposition Method

**DOI:** 10.3390/e21060597

**Published:** 2019-06-16

**Authors:** Hassan Khan, Rasool Shah, Poom Kumam, Muhammad Arif

**Affiliations:** 1 Department of Mathematics, Abdul Wali Khan University, Mardan 23200, Pakistan; 2 Center of Excellence in Theoretical and Computational Science (TaCS-CoE) & Department of Mathematics, Faculty of Science, King Mongkut’s University of Technology Thonburi (KMUTT), 126 Pracha Uthit Rd., Bang Mod, Thung Khru, Bangkok 10140, Thailand; 3 Department of Medical Research, China Medical University Hospital, China Medical University, Taichung 40402, Taiwan

**Keywords:** natural transform decomposition method, fractional-order heat and wave equations, Caputo operator

## Abstract

In the present article, fractional-order heat and wave equations are solved by using the natural transform decomposition method. The series form solutions are obtained for fractional-order heat and wave equations, using the proposed method. Some numerical examples are presented to understand the procedure of natural transform decomposition method. The natural transform decomposition method procedure has shown that less volume of calculations and a high rate of convergence can be easily applied to other nonlinear problems. Therefore, the natural transform decomposition method is considered to be one of the best analytical techniques, in order to solve fractional-order linear and nonlinear Partial deferential equations, particularly fractional-order heat and wave equation.

## 1. Introduction

The idea of entropy and fractional calculus are attractive, further prevalent for investigating the dynamics of complex schemes. In recent years, fractional calculus (FC) has been progressively applied in various fields of science. Natural development identified with viscoelasticity, models of porous electrodes, thermal stresses, electromagnetism, propagation of energy in dissipative systems, relaxation vibrations and thermoelasticity are effectively portrayed by fractional differential equations (FDE’s) [1]. The knowledge of entropy was presented in the field of thermodynamics by Clausius (1862) and Boltzmann (1896) and was further applied by Shannon (1948) and Jaynes (1957) in information theory. Newly, more universal entropy measures have been suggested for applications in numerous varieties of complex systems outstanding for the relaxation of the additives axiom [2]. The concept of entropy for analyzing the dynamics of multi-particle systems with an integer and fractional order behavior. The entropy production rate for the fractional diffusion procedure was considered in [3,4]. In [5], it has been shown that the total spectral entropy can be used as a measure of the data comfortable in a fractional order model of anomalous diffusion. Entropies based on fractional calculus [6], integer and fractional dynamical systems can be solved by entropy analysis [7], nonlinear partial differential equations and third-order dispersive [8,9] in entropy and convexity. Bifurcation and recurrent analysis of memristive circuits [10], density analysis of multi-wing and multi-scroll chaotic systems [11], and numerical integration in a simulation of conservative chaotic systems [12]. Fractional derivative advection–diffusion in two-dimensional semi-conductor systems and the dynamics of a national soccer league [13]. The exact solution to differential equations (DEs) of fractional order with mixed partial derivatives [14] and space-fractional diffusion equation and Tsallis relative entropy [15].

Joseph Fourier first developed the heat equation in 1822. The heat equation is connected with the study of Brownian motion. This motion was solved by Robert Brown. The irregular movement of particles suspended in a liquid (gas or fluid) come about because of their impact with the quick moving atoms in the liquid. Heat is the dynamic energy of particles that are being exchanged. When emotional vitality is exchanged from increased surrendering to a cooler scheme, faster moving atoms in the environment crash into the dividers of the scheme that exchanges a portion of this energy to the atoms of the system and makes them move faster [16,17,18]. The wave equation is a significant second order linear partial differential equations (PDE’s) for the description of waves as they occur in traditional physics such as fluid dynamics, mechanical waves and the electromagnetic wave equation is an important PDE that arises in fields like acoustics, electromagnetics and fluid dynamics. They are light, sound, gravity and even matter (in relativistic quantum mechanics, the Klein–Gordon equation). The one and three-dimensional wave equation was discovered by Alembert and Euler. The solutions of heat and wave equations have attracted the attention of various authors in mathematics, such as the optimal homotopy asymptotic method (OHAM) [19], the modified Adomian decomposition method (MADM) [20], the variational iteration method [21], the differential transform method (DTM) [22], the homotopy perturbation method (HPM), [23], Bernstein polynomials with the operational matrix [24], Elzaki transform and the projected differential transform method for nonlinear wave equations [25], the variational iteration method with the help of the Yang–Laplace transform fractional heat equation [26], and the Aboodh decomposition method [27].

In the present work, we are applying the natural transform decomposition method (NTDM), to solve the following types of fractional partial differential equations (FPDE’s).

$$\frac{\partial^{\gamma }\upsilon }{\partial t^{\gamma }}=g\left(x,y,z\right)\upsilon_{x,x}+h\left(x,y,z\right)\upsilon_{y,y}+k\left(x,y,z\right)\upsilon_{z,z}$$

with initial condition

$$\upsilon \left(x,y,z,0\right)=u\left(x,y,z\right),\;\upsilon_{t}\left(x,y,z,,0\right)=p\left(x,y,z\right).$$

Natural transform and Adomian decomposition methods are two powerful methods that have been used to develop the natural transform decomposition method. Many physical phenomena which are modeled by PDE and FPDEs are solved by using NTDM, such as the analytical solution of a couple of systems of nonlinear PDE’s is suggested in [28], the solution nonlinear ODE’s are successfully presented in [29], nonlinear PDEs [30], fractional unsteady flow of a polytropic gas model [31], fractional telegraph equations [32], fractional Fokker–Plank equation and Schrödinger equation [33]. The accuracy of the proposed method is compared with the solutions obtained by HPM and Modified homotopy perturbation method (MHPM). The comparisons has shown that the proposed has a higher rate of convergence than HPM and MHPM. The rest of the article is structured as: in Section 2, we recall several basic properties and definitions from natural transform and fractional calculus. In Section 3, we present the idea of the natural transform decomposition method. In Section 4, we explain many problems with maintaining the accuracy and efficiency of the proposed method, while the last section is devoted to conclusions.

## 2. Preliminaries

**Definition 1.**  *The natural transform of g(t) is defined as [34,35]:*

$$N^{+}\left[g\left(t\right)\right]=Q\left(s,u\right)=\frac{1}{u}\int_{0}^{\infty }e^{\frac{-st}{u}}g\left(t\right)dt;\;s,u>0,$$

*where s and u are the transform variables.*

**Definition 2.**  *The inverse natural transform of a function is defined by*

$$N^{-}\left[Q\left(s,u\right)\right]=g\left(t\right)=\frac{1}{2\pi i}\int_{p-i\infty }^{p+i\infty }e^{\frac{st}{u}}Q(s,u)ds,$$

*where s and u are the natural transform variables and p is a real constant and the integral is taken along s=p in the complex plane s=x+iy.*

**Definition 3.**  *Natural Transform of nth Derivative*

*If $g^{n}\left(t\right)$ is the nth derivative of function g(t) is given by*

$$N\left[g^{n}\left(t\right)\right]=Q_{n}\left(s,u\right)=\frac{s^{n}}{u^{n}}Q\left(s,u\right)-\sum_{k=0}^{n-1}\frac{s^{n-(k+1)}}{u^{n-k}}g^{k}\left(0\right),\;n\geq 1.$$

**Theorem 1.**  *If H(s,u), L(s,u) are the natural transform of respective functions h(t) and l(t) both defined in set A, then*

N[h∗l]=uH(s,u)L(s,u),

*where h∗l is convolution of two functions h and l.*

**Definition 4.**  *The Riemann–Liouville fractional integral*

$$I_{x}^{\gamma }g\left(x\right)=\left\{\begin{array}{cc} g(x) & if\;\gamma =0,\\ \frac{1}{\Gamma (\gamma )}\int_{0}^{x}{\left(x-\upsilon \right)}^{\gamma -1}g\left(\upsilon \right)d\upsilon & if\;\gamma >0, \end{array}\right.$$

*where *Γ* denotes the gamma function defined by*

$$\Gamma \left(\omega \right)=\int_{0}^{\infty }e^{-x}x^{\omega -1}dx\;\omega \in C.$$

*In this study, Caputo et al. suggested a revised fractional derivative operator in order to overcome inconsistency measured in the Riemann–Liouville derivative. The above mathematical statement described a Caputo fractional derivative operator of initial and boundary conditions for fractional as well as integer order derivatives [36,37].*

**Definition 5.**  *The Caputo operator of order γ for a fractional derivative is given by the following mathematical expression for n∈N, x>0, $g\in C_{t}$, t≥−1 [38]:*

$$D^{\gamma }g\left(x\right)=\frac{\partial^{\gamma }g\left(x\right)}{\partial t^{\gamma }}=\left\{\begin{array}{cc} I^{n-\gamma }\left[\frac{\partial^{\gamma }g\left(x\right)}{\partial t^{\gamma }}\right], & if\;n-1<\gamma \leq n,\;n\in N\\ \frac{\partial^{\gamma }g\left(x\right)}{\partial t^{\gamma }}. \end{array}\right.$$

## 3. Idea of the Fractional Natural Transform Decomposition Method

In this section, the natural transform decomposition method to find the general solution fractional-order equations:

(1) $$D^{\gamma }\upsilon \left(x,t\right)+L\upsilon \left(x,t\right)+N\upsilon \left(x,t\right)=q\left(x,t\right),\;x,t\geq 0,\;m-1<\gamma <m,$$

where $D^{\gamma }=\frac{\partial^{\gamma }}{\partial t^{\gamma }}$ the Caputo Operator γ,m∈N, where L and N are linear and nonlinear functions, and *q* is the source function.

The initial condition is

(2) υ(x,0)=k(x).

Applying the natural transform to Equation (1), we have

(3) $$N^{+}\left[D^{\gamma }\upsilon \left(x,t\right)\right]+N^{+}\left[L\upsilon (x,t)+N\upsilon (x,t)\right]=N^{+}\left[q(x,t)\right],$$

and using the differentiation property of natural transform, we get

$$\frac{s^{\gamma }}{u^{\gamma }}N^{+}\left[\upsilon (x,t)\right]-\frac{s^{\gamma -1}}{u^{\gamma }}\upsilon \left(x,0\right)=N^{+}\left[q(x,t)\right]-N^{+}\left[L\upsilon (x,t)+N\upsilon (x,t)\right],$$

(4) $$N^{+}\left[\upsilon (x,t)\right]=\frac{k(x)}{s}+\frac{u^{\gamma }}{s^{\gamma }}N^{+}\left[q(x,t)\right]-\frac{u^{\gamma }}{s^{\gamma }}N^{+}\left[L\upsilon (x,t)+N\upsilon (x,t)\right].$$

The NTDM solution υ(x,t) is represented by the following infinite series:

(5) $$\upsilon \left(x,t\right)=\sum_{j=0}^{\infty }\upsilon_{j}\left(x,t\right),$$

and the nonlinear terms (if any) in the problem are defined by the infinite series of Adomian polynomials,

(6) $$N\upsilon (x,t)=\sum_{j=0}^{\infty }A_{j},$$

(7) $$A_{j}=\frac{1}{j!}{\left[\frac{d^{j}}{d\lambda^{j}}\left[N\sum_{j=0}^{\infty }\left(\lambda^{j}\upsilon_{j}\right)\right]\right]}_{\lambda =0},\;j=0,1,2\ldots$$

substitution Equation (5) and Equation (6) in Equation (4), we get

(8) $$N^{+}\left[\sum_{j=0}^{\infty }\upsilon \left(x,t\right)\right]=\frac{k(x)}{s}+\frac{u^{\gamma }}{s^{\gamma }}N^{+}\left[q(x,t)\right]-\frac{u^{\gamma }}{s^{\gamma }}N^{+}\left[L\sum_{j=0}^{\infty }\upsilon_{j}\left(x,t\right)+\sum_{j=0}^{\infty }A_{j}\right].$$

Applying the linearity of the natural transform,

$$N^{+}\left[\upsilon_{0}\left(x,t\right)\right]=\frac{\upsilon (x,0)}{s}+\frac{u^{\gamma }}{s^{\gamma }}N^{+}\left[q(x,t)\right],$$

$$N^{+}\left[\upsilon_{1}\left(x,t\right)\right]=-\frac{u^{\gamma }}{s^{\gamma }}N^{+}\left[L\upsilon_{0}\left(x,t\right)+A_{0}\right].$$

Generally, we can write

(9) $$N^{+}\left[\upsilon_{j+1}\left(x,t\right)\right]=-\frac{u^{\gamma }}{s^{\gamma }}N^{+}\left[L\upsilon_{j}\left(x,t\right)+A_{j}\right],\;j\geq 1.$$

Applying the inverse natural transform, in Equation (9),

$$\upsilon_{0}\left(x,t\right)=k\left(x,t\right),$$

(10) $$\upsilon_{j+1}\left(x,t\right)=-N^{-}\left[\frac{u^{\gamma }}{s^{\gamma }}N^{+}\left[L\upsilon_{j}\left(x,t\right)+A_{j}\right]\right].$$

## 4. Results

**Example 1.**  *Consider the one-dimensional fractional heat equation [19]:*

(11) $$\frac{\partial^{\gamma }\upsilon }{\partial t^{\gamma }}-\frac{x^{2}}{2}\frac{\partial^{2}\upsilon }{\partial x^{2}}=0,\;0<\gamma \leq 1,\;t\geq 0,$$

*with initial condition*

(12) $$\upsilon \left(x,0\right)=x^{2}.$$

*Taking the natural transform of Equation (11),*

$$\frac{s^{\gamma }}{u^{\gamma }}N^{+}\left[\upsilon (x,t)\right]-\frac{s^{\gamma -1}}{u^{\gamma }}\upsilon (x,0)=N^{+}\left[\frac{x^{2}}{2}\frac{\partial^{2}\upsilon }{\partial x^{2}}\right].$$

*Applying inverse natural transform,*

$$\upsilon (x,t)=N^{-}\left[\frac{\upsilon (x,0)}{s}-\frac{u^{\gamma }}{s^{\gamma }}N^{+}\left[\frac{x^{2}}{2}\frac{\partial^{2}\upsilon }{\partial x^{2}}\right]\right].$$

*Using the ADM procedure, we get*

$$\upsilon_{0}\left(x,t\right)=N^{-}\left[\frac{\upsilon (x,0)}{s}\right]=N^{-}\left[\frac{x^{2}}{s}\right],$$

(13) $$\upsilon_{0}\left(x,t\right)=x^{2},$$

$$\upsilon_{j+1}\left(x,t\right)=N^{-}\left[\frac{u^{\gamma }}{s^{\gamma }}N^{+}\left[\frac{x^{2}}{2}\frac{\partial^{2}\upsilon_{j}}{\partial x^{2}}\right]\right],\;j=0,1,2,\ldots$$

*for j=0*

(14) $$\begin{array}{c} \upsilon_{1}\left(x,t\right)=N^{-}\left[\frac{u^{\gamma }}{s^{\gamma }}N^{+}\left[\frac{x^{2}}{2}\frac{\partial^{2}\upsilon_{0}}{\partial x^{2}}\right]\right],\\ \upsilon_{1}\left(x,t\right)=N^{-}\left[\frac{x^{2}u^{\gamma }}{s^{\gamma +1}}\right]=x^{2}\frac{t^{\gamma }}{\Gamma (\gamma +1)}. \end{array}$$

*The subsequent terms are*

(15) $$\begin{array}{c} \upsilon_{2}\left(x,t\right)=N^{-}\left[\frac{u^{\gamma }}{s^{\gamma }}N^{+}\left[\frac{x^{2}}{2}\frac{\partial^{2}\upsilon_{1}}{\partial x^{2}}\right]\right]=x^{2}\frac{t^{2\gamma }}{\Gamma (2\gamma +1)},\\ \upsilon_{3}\left(x,t\right)=N^{-}\left[\frac{u^{\gamma }}{s^{\gamma }}N^{+}\left[\frac{x^{2}}{2}\frac{\partial^{2}\upsilon_{2}}{\partial x^{2}}\right]\right]=x^{2}\frac{t^{3\gamma }}{\Gamma (3\gamma +1)},\\ \upsilon_{4}\left(x,t\right)=N^{-}\left[\frac{u^{\gamma }}{s^{\gamma }}N^{+}\left[\frac{x^{2}}{2}\frac{\partial^{2}\upsilon_{3}}{\partial x^{2}}\right]\right]=x^{2}\frac{t^{4\gamma }}{\Gamma (4\gamma +1)},\\ .\\ . \end{array}$$

*The NTDM solution for Example 1 is*

$$\upsilon \left(x,t\right)=\upsilon_{0}\left(x,t\right)+\upsilon_{1}\left(x,t\right)+\upsilon_{2}\left(x,t\right)+\upsilon_{3}\left(x,t\right)+\upsilon_{4}\left(x,t\right)\cdots$$

$$\upsilon \left(x,t\right)=x^{2}\left(1+\frac{t^{\gamma }}{\Gamma (\gamma +1)}+\frac{t^{2\gamma }}{\Gamma (2\gamma +1)}+\frac{t^{3\gamma }}{\Gamma (3\gamma +1)}+\frac{t^{4\gamma }}{\Gamma (4\gamma +1)}\cdots \right),$$

*when γ=1, then the NTDM solution is*

(16) $$\upsilon \left(x,t\right)=x^{2}\left(1+t+\frac{t^{2}}{2!}+\frac{t^{3}}{3!}+\frac{t^{4}}{4!}\cdots \right).$$

*This result is calculated to get the exact solution in a closed form:*

$$\upsilon \left(x,t\right)=x^{2}e^{t}.$$

**Figure 1:**
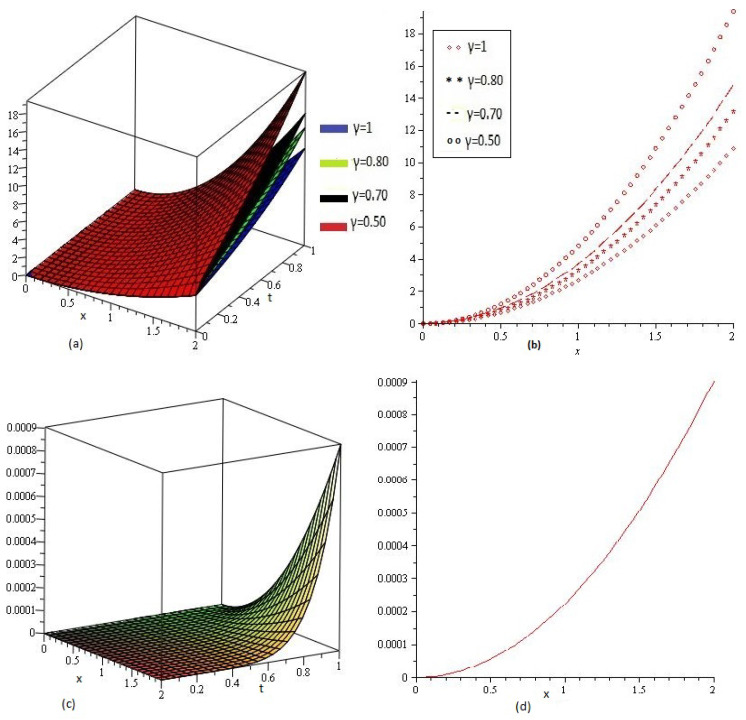
(**a**) Comparison with numerical solution of υ(x,t) by NTDM of Example 1, for different values of γ and (**b**) for t=1; (**c**) and (**d**) are error plots of Example 1.

**Figure 2:**
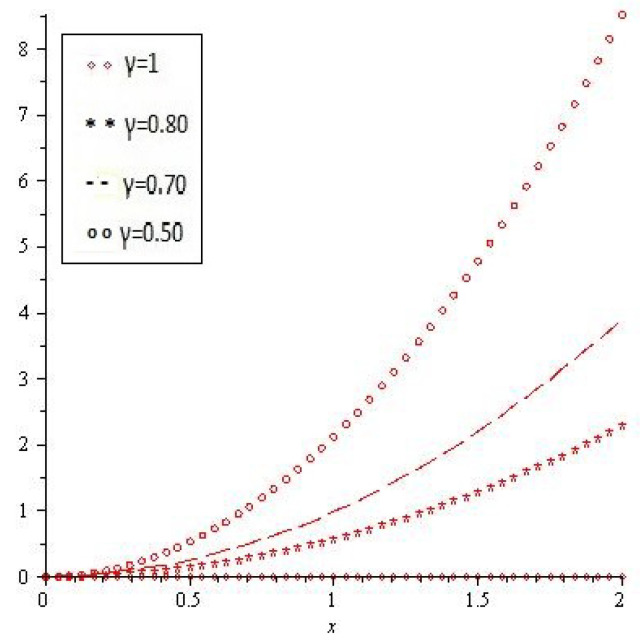
Error plot of Example 1 for different values of γ.

Figure 1a,b shows the behavior of obtained solution υ(x,t) by the proposed method for different values of γ=1, 0.80, 0.70, 0.50 and t=1, then Figure 1c,d are error plots for γ=1. Figure 2 combine error plots the range of *x* 0<x≤2.

**Example 2.**  *Consider the two-dimensional fractional heat equation [19]:*

(17) $$\frac{\partial^{\gamma }\upsilon }{\partial t^{\gamma }}-\frac{y^{2}}{2}\frac{\partial^{2}\upsilon }{\partial x^{2}}-\frac{x^{2}}{2}\frac{\partial^{2}\upsilon }{\partial y^{2}}=0,\;0<\gamma \leq 1,$$

*with initial condition*

(18) $$\upsilon \left(x,y,0\right)=y^{2}.$$

*Taking the natural transform of Equation (17),*

$$\frac{s^{\gamma }}{u^{\gamma }}N^{+}\left[\upsilon (x,y,t)\right]-\frac{s^{\gamma -1}}{u^{\gamma }}\upsilon (x,y,0)=N^{+}\left[\frac{y^{2}}{2}\frac{\partial^{2}\upsilon }{\partial x^{2}}+\frac{x^{2}}{2}\frac{\partial^{2}\upsilon }{\partial y^{2}}\right].$$

*Applying inverse natural transform,*

$$\upsilon (x,y,t)=N^{-}\left[\frac{\upsilon (x,y,0)}{s}+\frac{u^{\gamma }}{s^{\gamma }}N^{+}\left[\frac{y^{2}}{2}\frac{\partial^{2}\upsilon }{\partial x^{2}}+\frac{x^{2}}{2}\frac{\partial^{2}\upsilon }{\partial y^{2}}\right]\right].$$

*Using the ADM procedure, we get*

$$\upsilon_{0}\left(x,y,t\right)=N^{-}\left[\frac{\upsilon (x,y,0)}{s}\right]=N^{-}\left[\frac{y^{2}}{s}\right],$$

(19) $$\upsilon_{0}\left(x,y,t\right)=y^{2},$$

$$\sum_{j=0}^{\infty }\upsilon_{j+1}\left(x,y,t\right)=N^{-}\left[\frac{u^{\gamma }}{s^{\gamma }}N^{+}\left[\frac{y^{2}}{2}\sum_{j=0}^{\infty }\upsilon_{xxj}+\frac{x^{2}}{2}\sum_{j=0}^{\infty }\upsilon_{yyj}\right]\right],$$

*for j=0*

(20) $$\begin{array}{c} \upsilon_{1}\left(x,y,t\right)=N^{-}\left[\frac{u^{\gamma }}{s^{\gamma }}N^{+}\left[\frac{y^{2}}{2}\frac{\partial^{2}\upsilon_{0}}{\partial x^{2}}+\frac{x^{2}}{2}\frac{\partial^{2}\upsilon_{0}}{\partial y^{2}}\right]\right],\\ \upsilon_{1}\left(x,y,t\right)=x^{2}\frac{t^{\gamma }}{\Gamma (\gamma +1)}. \end{array}$$

*The subsequent terms are*

(21) $$\begin{array}{c} \upsilon_{2}\left(x,y,t\right)=N^{-}\left[\frac{u^{\gamma }}{s^{\gamma }}N^{+}\left[\frac{y^{2}}{2}\frac{\partial^{2}\upsilon_{1}}{\partial x^{2}}+\frac{x^{2}}{2}\frac{\partial^{2}\upsilon_{1}}{\partial y^{2}}\right]\right]=y^{2}\frac{t^{2\gamma }}{\Gamma (2\gamma +1)},\\ \upsilon_{3}\left(x,y,t\right)=N^{-}\left[\frac{u^{\gamma }}{s^{\gamma }}N^{+}\left[\frac{y^{2}}{2}\frac{\partial^{2}\upsilon_{2}}{\partial x^{2}}+\frac{x^{2}}{2}\frac{\partial^{2}\upsilon_{2}}{\partial y^{2}}\right]\right]=x^{2}\frac{t^{3\gamma }}{\Gamma (3\gamma +1)},\\ \upsilon_{4}\left(x,y,t\right)=N^{-}\left[\frac{u^{\gamma }}{s^{\gamma }}N^{+}\left[\frac{y^{2}}{2}\frac{\partial^{2}\upsilon_{3}}{\partial x^{2}}+\frac{x^{2}}{2}\frac{\partial^{2}\upsilon_{3}}{\partial y^{2}}\right]\right]=y^{2}\frac{t^{4\gamma }}{\Gamma (4\gamma +1)}.\\ .\\ . \end{array}$$

*The NTDM solution for Example 2 is*

$$\upsilon \left(x,y,t\right)=\upsilon_{0}\left(x,y,t\right)+\upsilon_{1}\left(x,y,t\right)+\upsilon_{2}\left(x,y,t\right)+\upsilon_{3}\left(x,y,t\right)+\upsilon_{4}\left(x,y,t\right)\cdots$$

$$\begin{array}{c} \upsilon \left(x,y,t\right)=x^{2}\left(\frac{t^{\gamma }}{\Gamma (\gamma +1)}+\frac{t^{3\gamma }}{\Gamma (3\gamma +1)}+\frac{t^{5\gamma }}{\Gamma (5\gamma +1)}+\frac{t^{7\gamma }}{\Gamma (7\gamma +1)}\cdots \right)\\ +y^{2}\left(1+\frac{t^{2\gamma }}{\Gamma (2\gamma +1)}+\frac{t^{4\gamma }}{\Gamma (4\gamma +1)}+\frac{t^{6\gamma }}{\Gamma (6\gamma +1)}\cdots \right). \end{array}$$

*This result is calculated to get the exact solution in a closed form:*

$$\upsilon \left(x,y,t\right)=x^{2}\sinh t+y^{2}\cosh t$$

**Figure 3:**
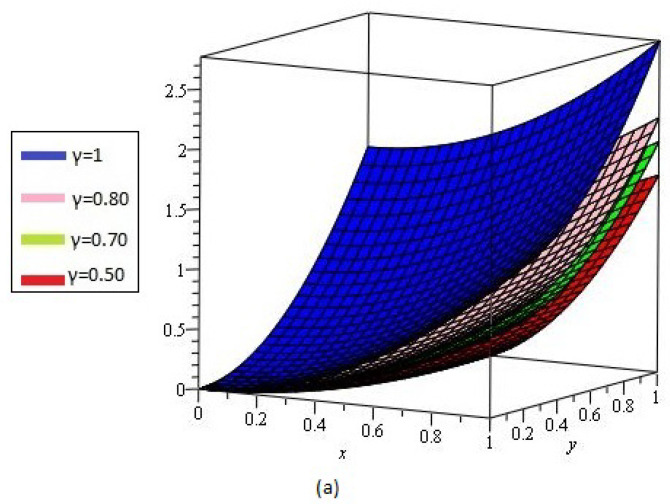

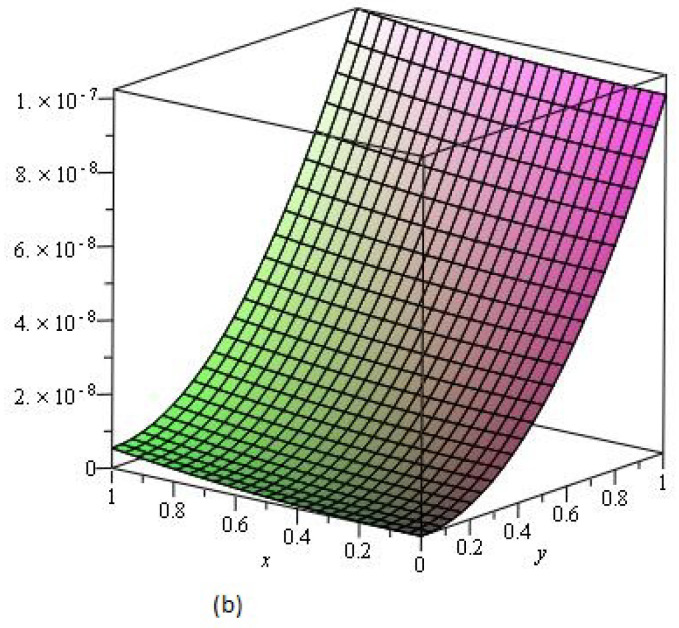
(**a**) Comparison with numerical solution of υ(x,y,t) by NTDM of Example 2, for different values of γ; (**b**) error plot for γ=1.

Figure 3a shows the behavior of obtained solution υ(x,y,t) by the proposed method for different values of γ=1, 0.80, 0.70, 0.50 and t=1; Figure 3b error plot for γ=1 the range of *x* and *y* 0<x,y≤1.

**Example 3.**  *Consider the three-dimensional fractional heat equation [19]:*

(22) $$\frac{\partial^{\gamma }\upsilon }{\partial t^{\gamma }}-{\left(xyz\right)}^{4}-\frac{1}{36}\left(x^{2}\frac{\partial^{2}\upsilon }{\partial x^{2}}+y^{2}\frac{\partial^{2}\upsilon }{\partial y^{2}}+z^{2}\frac{\partial^{2}\upsilon }{\partial z^{2}}\right),\;0<\gamma \leq 1,\;t\geq 0,$$

*with initial condition*

(23) υ(x,y,z,0)=0.

*Taking the natural transform of Equation (22),*

$$\frac{s^{\gamma }}{u^{\gamma }}N^{+}\left[\upsilon (x,y,z,t)\right]-\frac{s^{\gamma -1}}{u^{\gamma }}\upsilon (x,y,z,0)=N^{+}\left[{\left(xyz\right)}^{4}\right]+N^{+}\left[\frac{1}{36}\left(x^{2}\frac{\partial^{2}\upsilon }{\partial x^{2}}+y^{2}\frac{\partial^{2}\upsilon }{\partial y^{2}}+z^{2}\frac{\partial^{2}\upsilon }{\partial z^{2}}\right)\right].$$

*Applying inverse natural transform*

$$\begin{array}{c} \upsilon (x,y,z,t)=N^{-}\left[\frac{\upsilon (x,y,z,0)}{s}+\frac{u^{\gamma }}{s^{\gamma }}N^{+}\left[{\left(xyz\right)}^{4}\right]\right]\\ +N^{-}\left[\frac{u^{\gamma }}{s^{\gamma }}N^{+}\left[\frac{1}{36}\left(x^{2}\frac{\partial^{2}\upsilon }{\partial x^{2}}+y^{2}\frac{\partial^{2}\upsilon }{\partial y^{2}}+z^{2}\frac{\partial^{2}\upsilon }{\partial z^{2}}\right)\right]\right]. \end{array}$$

*Using the ADM procedure, we get*

$$\upsilon_{0}\left(x,y,z,t\right)=N^{-}\left[\frac{\upsilon (x,y,z,0)}{s}+\frac{u^{\gamma }}{s^{\gamma }}N^{+}\left[{\left(xyz\right)}^{4}\right]\right],$$

(24) $$\upsilon_{0}\left(x,y,z,t\right)=N^{-}\left(\frac{x^{4}y^{4}z^{4}}{s}\right)=x^{4}y^{4}z^{4}\frac{t^{\gamma }}{\Gamma (\gamma +1)},$$

$$\sum_{j=0}^{\infty }\left(x,y,z,t\right)=N^{-}\left[\frac{u^{\gamma }}{s^{\gamma }}N^{+}\left[\frac{1}{36}\left(x^{2}\sum_{j=0}^{\infty }\upsilon_{xj}+y^{2}\sum_{j=0}^{\infty }\upsilon_{yj}+z^{2}\sum_{j=0}^{\infty }\upsilon_{zj}\right)\right]\right],$$

*for j=0*

(25) $$\begin{array}{c} \upsilon_{1}\left(x,y,z,t\right)=N^{-}\left[\frac{u^{\gamma }}{s^{\gamma }}N^{+}\left[x^{2}\frac{\partial^{2}\upsilon_{0}}{\partial x^{2}}+y^{2}\frac{\partial^{2}\upsilon_{0}}{\partial y^{2}}+z^{2}\frac{\partial^{2}\upsilon_{0}}{\partial z^{2}}\right]\right],\\ \upsilon_{1}\left(x,y,z,t\right)=N^{-}\left[\frac{x^{4}y^{4}z^{4}u^{\gamma }}{s^{\gamma +1}}\right]=x^{4}y^{4}z^{4}\frac{t^{2\gamma }}{\Gamma (2\gamma +1)}. \end{array}$$

*The subsequent terms are*

(26) $$\begin{array}{c} \upsilon_{2}\left(x,y,z,t\right)=N^{-}\left[\frac{u^{\gamma }}{s^{\gamma }}N^{+}\left[x^{2}\frac{\partial^{2}\upsilon_{1}}{\partial x^{2}}+y^{2}\frac{\partial^{2}\upsilon_{1}}{\partial y^{2}}+z^{2}\frac{\partial^{2}\upsilon_{1}}{\partial z^{2}}\right]\right]=x^{4}y^{4}z^{4}\frac{t^{3\gamma }}{\Gamma (3\gamma +1)},\\ \upsilon_{3}\left(x,y,z,t\right)=N^{-}\left[\frac{u^{\gamma }}{s^{\gamma }}N^{+}\left[x^{2}\frac{\partial^{2}\upsilon_{1}}{\partial x^{2}}+y^{2}\frac{\partial^{2}\upsilon_{1}}{\partial y^{2}}+z^{2}\frac{\partial^{2}\upsilon_{1}}{\partial z^{2}}\right]\right]=x^{4}y^{4}z^{4}\frac{t^{4\gamma }}{\Gamma (4\gamma +1)},\\ \upsilon_{4}\left(x,y,z,t\right)=N^{-}\left[\frac{u^{\gamma }}{s^{\gamma }}N^{+}\left[x^{2}\frac{\partial^{2}\upsilon_{1}}{\partial x^{2}}+y^{2}\frac{\partial^{2}\upsilon_{1}}{\partial y^{2}}+z^{2}\frac{\partial^{2}\upsilon_{1}}{\partial z^{2}}\right]\right]=x^{4}y^{4}z^{4}\frac{t^{5\gamma }}{\Gamma (5\gamma +1)}.\\ .\\ .\\ . \end{array}$$

*The NTDM solution for Example 3 is*

$$\upsilon \left(x,y,z,t\right)=\upsilon_{0}\left(x,y,z,t\right)+\upsilon_{1}\left(x,y,z,t\right)+\upsilon_{2}\left(x,y,z,t\right)+\upsilon_{3}\left(x,y,z,t\right)+\cdots$$

$$\upsilon \left(x,y,z,t\right)=x^{4}y^{4}z^{4}\left(\frac{t^{\gamma }}{\Gamma (\gamma +1)}+\frac{t^{2\gamma }}{\Gamma (2\gamma +1)}+\frac{t^{3\gamma }}{\Gamma (3\gamma +1)}+\frac{t^{4\gamma }}{\Gamma (4\gamma +1)}\cdots \right),$$

*when γ=1, then the NTDM solution is*

(27) $$\upsilon \left(x,y,z,t\right)=x^{4}y^{4}z^{4}\left(t+\frac{t^{2}}{2!}+\frac{t^{3}}{3!}+\frac{t^{4}}{4!}\cdots \right).$$

*This result is calculated to get the exact solution in a closed form:*

$$\upsilon \left(x,y,z,t\right)=\left(e^{t}-1\right)x^{4}y^{4}z^{4}.$$

**Figure 4:**
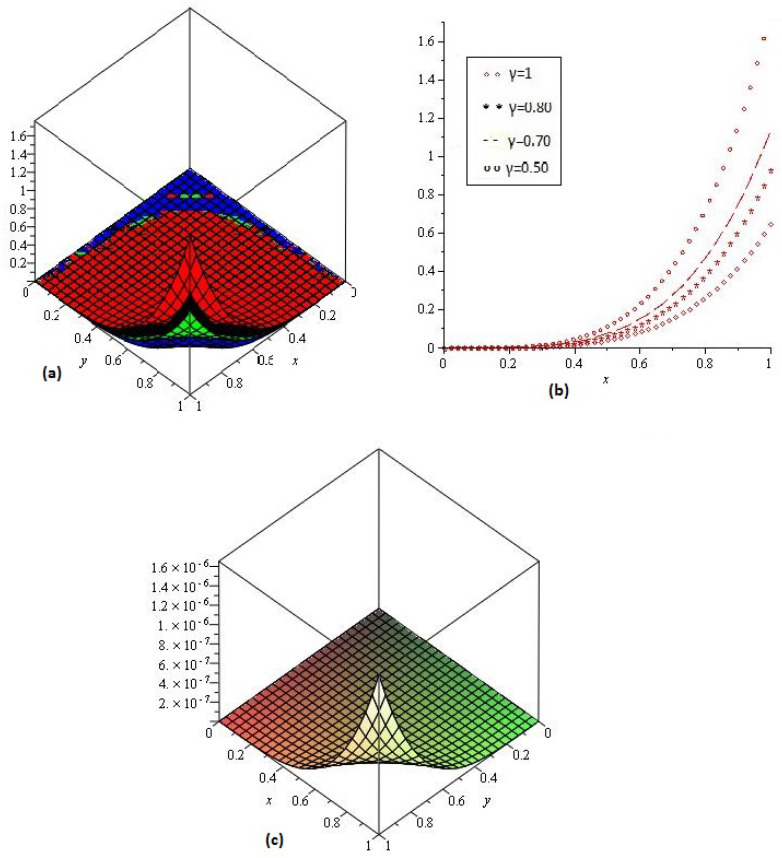
(**a**) Comparison with numerical solution of υ(x,t) by NTDM of Example 3, for different values of γ and (**b**) for t=1; (**c**) error plot of Example 3 for γ=1.

Figure 4a,b shows the behavior of obtained solution υ(x,y,z,t) by the proposed method for different values of γ=1, 0.80, 0.70, 0.50 and z,t=1; Figure 4c error plot for γ=1 the range of *x* and *y* 0<x,y≤1.

**Example 4.**  *Consider the one-dimensional fractional heat equation [19]:*

(28) $$\frac{\partial^{\gamma }\upsilon }{\partial t^{\gamma }}-\frac{x^{2}}{2}\frac{\partial^{2}\upsilon }{\partial x^{2}}=0,\;0<\gamma \leq 2,\;t\geq 0,$$

*with initial condition*

(29) $$\upsilon \left(x,0\right)=x,\;\upsilon_{t}\left(x,0\right)=x^{2}.$$

*Taking natural transform of Equation (28),*

$$\frac{s^{\gamma }}{u^{\gamma }}N^{+}\left[\upsilon (x,t)\right]-\frac{s^{\gamma -1}}{u^{\gamma }}\upsilon (x,0)-\frac{s^{\gamma -2}}{u^{\gamma -1}}\upsilon_{t}\left(x,0\right)=N^{+}\left[\frac{x^{2}}{2}\frac{\partial^{2}\upsilon }{\partial x^{2}}\right].$$

*Applying inverse natural transform,*

$$\upsilon (x,t)=N^{-}\left[\frac{\upsilon (x,0)}{s}+\frac{u}{s}\upsilon_{t}\left(x,0\right)-\frac{u^{\gamma }}{s^{\gamma }}N^{+}\left[\frac{x^{2}}{2}\frac{\partial^{2}\upsilon }{\partial x^{2}}\right]\right].$$

*Using the ADM procedure, we get*

$$\upsilon_{0}\left(x,t\right)=N^{-}\left[\frac{\upsilon (x,0)}{s}+\frac{u}{s}\upsilon_{t}\left(x,0\right)\right]=N^{-}\left[x\frac{1}{s}+x^{2}\frac{u}{s}\right],$$

(30) $$\upsilon_{0}\left(x,t\right)=x+x^{2}t,$$

$$\upsilon_{j+1}\left(x,t\right)=N^{-}\left[\frac{u^{\gamma }}{s^{\gamma }}N^{+}\left[\frac{x^{2}}{2}\frac{\partial^{2}\upsilon_{j}}{\partial x^{2}}\right]\right],\;j=0,1,2,\cdots$$

*for j=0*

(31) $$\begin{array}{c} \upsilon_{1}\left(x,t\right)=N^{-}\left[\frac{u^{\gamma }}{s^{\gamma }}N^{+}\left[\frac{x^{2}}{2}\frac{\partial^{2}\upsilon_{0}}{\partial x^{2}}\right]\right],\\ \upsilon_{1}\left(x,t\right)=N^{-}\left[\frac{x^{2}u^{\gamma }}{s^{\gamma +2}}\right]=x^{2}\frac{t^{\gamma +1}}{\Gamma (\gamma +2)}. \end{array}$$

*The subsequent terms are*

(32) $$\begin{array}{c} \upsilon_{2}\left(x,t\right)=N^{-}\left[\frac{u^{\gamma }}{s^{\gamma }}N^{+}\left[\frac{x^{2}}{2}\frac{\partial^{2}\upsilon_{1}}{\partial x^{2}}\right]\right]=x^{2}\frac{t^{2\gamma +1}}{\Gamma (2\gamma +2)},\\ \upsilon_{3}\left(x,t\right)=N^{-}\left[\frac{u^{\gamma }}{s^{\gamma }}N^{+}\left[\frac{x^{2}}{2}\frac{\partial^{2}\upsilon_{2}}{\partial x^{2}}\right]\right]=x^{2}\frac{t^{3\gamma +1}}{\Gamma (3\gamma +3)},\\ \upsilon_{4}\left(x,t\right)=N^{-}\left[\frac{u^{\gamma }}{s^{\gamma }}N^{+}\left[\frac{x^{2}}{2}\frac{\partial^{2}\upsilon_{3}}{\partial x^{2}}\right]\right]=x^{2}\frac{t^{4\gamma +1}}{\Gamma (4\gamma +4)},\\ .\\ . \end{array}$$

*The NTDM solution for Example 4 is*

$$\upsilon \left(x,t\right)=\upsilon_{0}\left(x,t\right)+\upsilon_{1}\left(x,t\right)+\upsilon_{2}\left(x,t\right)+\upsilon_{3}\left(x,t\right)+\upsilon_{4}\left(x,t\right)\cdots$$

$$\upsilon \left(x,t\right)=x+x^{2}\left(t+\frac{t^{\gamma +1}}{\Gamma (\gamma +2)}+\frac{t^{2\gamma +1}}{\Gamma (2\gamma +2)}+\frac{t^{3\gamma +1}}{\Gamma (3\gamma +2)}+\frac{t^{4\gamma +1}}{\Gamma (4\gamma +2)}\cdots \right),$$

*when γ=2, then NTDM solution is*

(33) $$\upsilon \left(x,t\right)=x+x^{2}\left(t+\frac{t^{3}}{3!}+\frac{t^{5}}{5!}+\frac{t^{7}}{7!}\cdots \right).$$

*This result is calculated to get the exact solution in a closed form:*

$$\upsilon \left(x,t\right)=x+x^{2}\sinh t$$

**Figure 5:**
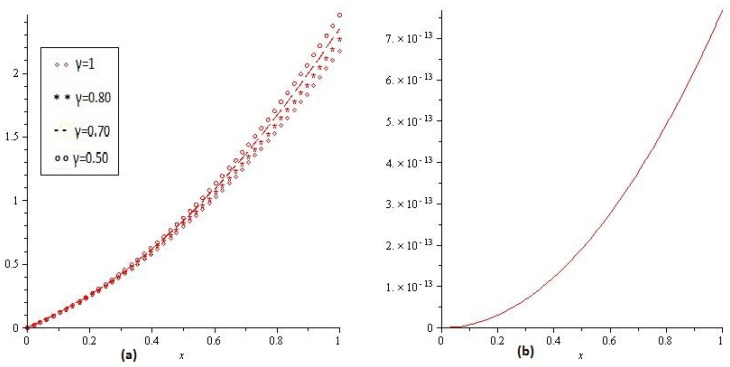
(**a**) Comparison with numerical solution of υ(x,t) by NTDM of Example 4, for different values of γ; (**b**) error plot for γ=2.

Figure 5a show the behavior of obtained solution υ(x,t) by the proposed method for different values of γ=2, 1.80, 1.70, 1.50 and t=1; Figure 5b error plot for γ=2 the range of *x* 0<x≤1.

**Example 5.**  *Consider the two-dimensional fractional wave equation [19]:*

(34) $$\frac{\partial^{\gamma }\upsilon }{\partial t^{\gamma }}-\frac{y^{2}}{12}\frac{\partial^{2}\upsilon }{\partial x^{2}}-\frac{x^{2}}{12}\frac{\partial^{2}\upsilon }{\partial y^{2}}=0,\;0<\gamma \leq 2,$$

*with initial condition*

(35) $$\upsilon \left(x,y,0\right)=x^{4},\;\upsilon_{t}\left(x,y,0\right)=y^{4}$$

*Taking natural transform of Equation (34),*

$$\frac{s^{\gamma }}{u^{\gamma }}N^{+}\left[\upsilon (x,y,t)\right]-\frac{s^{\gamma -1}}{u^{\gamma }}\upsilon (x,y,0)-\frac{s^{\gamma -2}}{u^{\gamma -1}}\upsilon_{t}\left(x,y,0\right)=N^{+}\left[\frac{y^{2}}{12}\frac{\partial^{2}\upsilon }{\partial x^{2}}+\frac{x^{2}}{12}\frac{\partial^{2}\upsilon }{\partial y^{2}}\right].$$

*Applying inverse natural transform*

$$\upsilon (x,y,t)=N^{-}\left[\frac{\upsilon (x,y,0)}{s}+\frac{u\upsilon_{t}\left(x,y,0\right)}{s^{2}}+\frac{u^{\gamma }}{s^{\gamma }}N^{+}\left[\frac{y^{2}}{12}\frac{\partial^{2}\upsilon }{\partial x^{2}}+\frac{x^{2}}{12}\frac{\partial^{2}\upsilon }{\partial y^{2}}\right]\right].$$

*Using the ADM procedure, we get*

$$\upsilon_{0}\left(x,y,t\right)=N^{-}\left[\frac{\upsilon (x,y,0)}{s}+\frac{u\upsilon_{t}\left(x,y,0\right)}{s^{2}}\right]=N^{-}\left[\frac{x^{4}}{s}+\frac{uy^{4}}{s^{2}}\right]$$

(36) $$\upsilon_{0}\left(x,y,t\right)=x^{4}+y^{4}t$$

$$\sum_{j=0}^{\infty }\upsilon_{j+1}\left(x,y,t\right)=N^{-}\left[\frac{u^{\gamma }}{s^{\gamma }}N^{+}\left[\frac{y^{2}}{12}\sum_{j=0}^{\infty }\upsilon_{xxj}+\frac{x^{2}}{12}\sum_{j=0}^{\infty }\upsilon_{yyj}\right]\right],$$

*for j=0*

(37) $$\begin{array}{c} \upsilon_{1}\left(x,y,t\right)=N^{-}\left[\frac{u^{\gamma }}{s^{\gamma }}N^{+}\left[\frac{y^{2}}{12}\frac{\partial^{2}\upsilon_{0}}{\partial x^{2}}+\frac{x^{2}}{12}\frac{\partial^{2}\upsilon_{0}}{\partial y^{2}}\right]\right]\\ \upsilon_{1}\left(x,y,t\right)=x^{4}\frac{t^{\gamma }}{\Gamma (\gamma +1)}+y^{4}\frac{t^{\gamma +1}}{\Gamma (\gamma +2)}. \end{array}$$

*The subsequent terms are*

(38) $$\begin{array}{c} \upsilon_{2}\left(x,y,t\right)=N^{-}\left[\frac{u^{\gamma }}{s^{\gamma }}N^{+}\left[\frac{y^{2}}{12}\frac{\partial^{2}\upsilon_{1}}{\partial x^{2}}+\frac{x^{2}}{12}\frac{\partial^{2}\upsilon_{1}}{\partial y^{2}}\right]\right]=x^{4}\frac{t^{2\gamma }}{\Gamma (2\gamma +1)}+y^{4}\frac{t^{2\gamma +1}}{\Gamma (2\gamma +2)},\\ \upsilon_{3}\left(x,y,t\right)=N^{-}\left[\frac{u^{\gamma }}{s^{\gamma }}N^{+}\left[\frac{y^{2}}{12}\frac{\partial^{2}\upsilon_{2}}{\partial x^{2}}+\frac{x^{2}}{12}\frac{\partial^{2}\upsilon_{2}}{\partial y^{2}}\right]\right]=x^{4}\frac{t^{3\gamma }}{\Gamma (3\gamma +1)}+y^{4}\frac{t^{3\gamma +1}}{\Gamma (3\gamma +2)},\\ \upsilon_{4}\left(x,y,t\right)=N^{-}\left[\frac{u^{\gamma }}{s^{\gamma }}N^{+}\left[\frac{y^{2}}{12}\frac{\partial^{2}\upsilon_{3}}{\partial x^{2}}+\frac{x^{2}}{12}\frac{\partial^{2}\upsilon_{3}}{\partial y^{2}}\right]\right]=y^{4}\frac{t^{4\gamma }}{\Gamma (4\gamma +1)}++y^{4}\frac{t^{4\gamma +1}}{\Gamma (4\gamma +2)},\\ .\\ . \end{array}$$

*The NTDM solution for Example 5 is*

$$\upsilon \left(x,y,t\right)=\upsilon_{0}\left(x,y,t\right)+\upsilon_{1}\left(x,y,t\right)+\upsilon_{2}\left(x,y,t\right)+\upsilon_{3}\left(x,y,t\right)+\upsilon_{4}\left(x,y,t\right)\cdots$$

$$\begin{array}{c} \upsilon \left(x,y,t\right)=x^{4}\left(1+\frac{t^{\gamma }}{\Gamma (\gamma +1)}+\frac{t^{2\gamma }}{\Gamma (2\gamma +1)}+\frac{t^{3\gamma }}{\Gamma (3\gamma +1)}+\frac{t^{4\gamma }}{\Gamma (4\gamma +1)}\cdots \right)\\ +y^{2}\left(1+\frac{t^{\gamma +1}}{\Gamma (\gamma +2)}+\frac{t^{2\gamma +1}}{\Gamma (2\gamma +2)}+\frac{t^{3\gamma +1}}{\Gamma (3\gamma +2)}+\frac{t^{4\gamma +1}}{\Gamma (4\gamma +2)}\cdots \right), \end{array}$$

*This result is calculated to get the exact solution in a closed form:*

$$\upsilon \left(x,y,t\right)=x^{4}\cosh t+y^{4}\sinh t.$$

**Figure 6:**
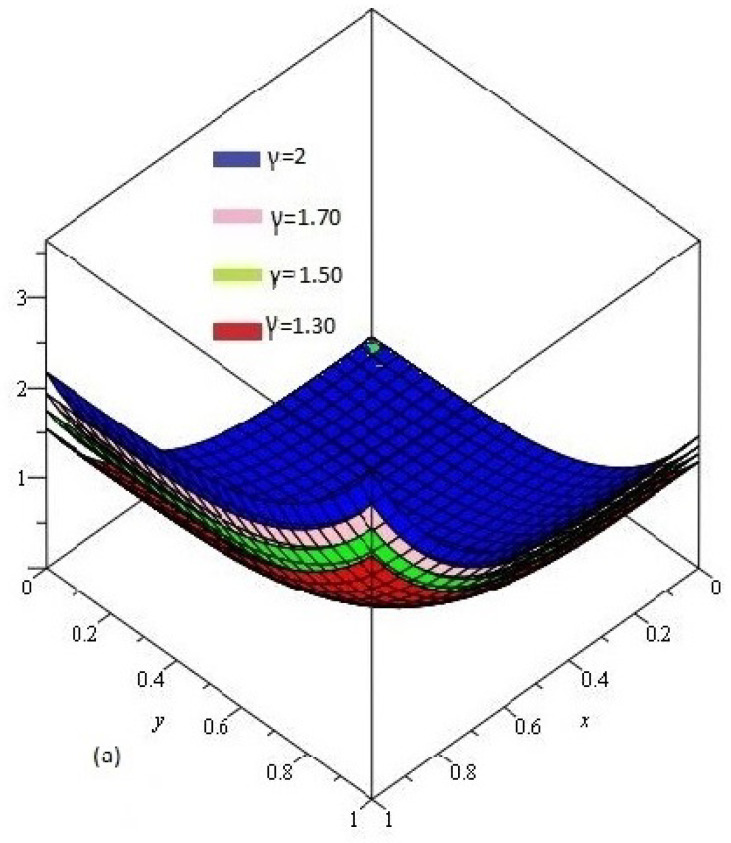

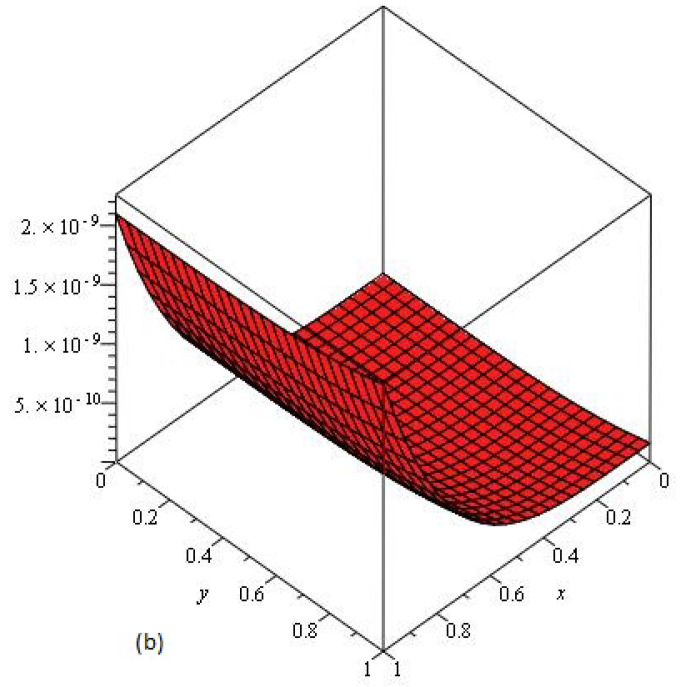
(**a**) Comparison with numerical solution of υ(x,y,t) by NTDM of Example 5, for different values of γ; (**b**) error plot for γ=2.

Figure 6a shows the behavior of obtained solution υ(x,y,t) by the proposed method for different values of γ=2, 1.80, 1.70, 1.50 and t=1; Figure 6b error plot for γ=2 the range of *x* and *y* 0<x,y≤1.

**Example 6.**  *Consider the three-dimensional fractional wave equation [19]:*

(39) $$\frac{\partial^{\gamma }\upsilon }{\partial t^{\gamma }}-\left(x^{2}+y^{2}+z^{2}\right)-\frac{1}{2}\left(x^{2}\frac{\partial^{2}\upsilon }{\partial x^{2}}+y^{2}\frac{\partial^{2}\upsilon }{\partial y^{2}}+z^{2}\frac{\partial^{2}\upsilon }{\partial z^{2}}\right)=0,\;0<\gamma \leq 2,\;t\geq 0,$$

*with initial condition*

(40) $$\upsilon \left(x,y,z,0\right)=0,\;\upsilon_{t}\left(x,y,z,0\right)=x^{2}+y^{2}-z^{2}.$$

*Taking natural transform of Equation (39),*

$$\begin{array}{c} \frac{s^{\gamma }}{u^{\gamma }}N^{+}\left[\upsilon (x,y,z,t)\right]-\frac{s^{\gamma -1}}{u^{\gamma }}\upsilon (x,y,z,0)-\frac{s^{\gamma -2}}{u^{\gamma -1}}\upsilon_{t}\left(x,y,z,0\right)\\ =N^{+}\left[x^{2}+y^{2}+z^{2}+\frac{1}{2}\left(x^{2}\frac{\partial^{2}\upsilon }{\partial x^{2}}+y^{2}\frac{\partial^{2}\upsilon }{\partial y^{2}}+z^{2}\frac{\partial^{2}\upsilon }{\partial z^{2}}\right)\right]. \end{array}$$

*Applying inverse natural transform,*

$$\begin{array}{c} \upsilon (x,y,z,t)=N^{-}\left[\frac{\upsilon (x,y,z,0)}{s}+\frac{u}{s^{2}}\left[\upsilon_{t}\left(x,y,z,0\right)\right]\right]\\ +N^{-}\left[\frac{u^{\gamma }}{s^{\gamma }}N^{+}\left[x^{2}+y^{2}+z^{2}+\frac{u^{\gamma }}{s^{\gamma }}N^{+}\left[\frac{1}{2}\left(x^{2}\frac{\partial^{2}\upsilon }{\partial x^{2}}+y^{2}\frac{\partial^{2}\upsilon }{\partial y^{2}}+z^{2}\frac{\partial^{2}\upsilon }{\partial z^{2}}\right)\right]\right]\right]. \end{array}$$

*Using the ADM procedure, we get*

$$\upsilon_{0}\left(x,y,z,t\right)=N^{-}\left[\frac{\upsilon (x,y,z,0)}{s}+\frac{u}{s^{2}}\left(\upsilon_{t}\left(x,y,z,0\right)\right)\right]$$

(41) $$\upsilon_{0}\left(x,y,z,t\right)=N^{-}\left(\frac{u(x^{2}+y^{2}-z^{2})}{s^{2}}\right)=\left(x^{2}+y^{2}-z^{2}\right)t$$

$$\sum_{j=0}^{\infty }\left(x,y,z,t\right)=N^{-}\left[\frac{u^{\gamma }}{s^{\gamma }}N^{+}\left[\frac{1}{2}\left(x^{2}\sum_{j=0}^{\infty }\upsilon_{xj}+y^{2}\sum_{j=0}^{\infty }\upsilon_{yj}+z^{2}\sum_{j=0}^{\infty }\upsilon_{zj}\right)\right]\right],$$

*for j=0*

(42) $$\begin{array}{c} \upsilon_{1}\left(x,y,z,t\right)=N^{-}\left[\frac{u^{\gamma }}{s^{\gamma }}N^{+}\left[x^{2}\frac{\partial^{2}\upsilon_{0}}{\partial x^{2}}+y^{2}\frac{\partial^{2}\upsilon_{0}}{\partial y^{2}}+z^{2}\frac{\partial^{2}\upsilon_{0}}{\partial z^{2}}\right]\right]\\ \upsilon_{1}\left(x,y,z,t\right)=x^{2}\frac{t^{\gamma }}{\Gamma (\gamma +1)}+x^{2}\frac{t^{\gamma +1}}{\Gamma (\gamma +2)}+y^{2}\frac{t^{\gamma }}{\Gamma (\gamma +1)}+y^{2}\frac{t^{\gamma +1}}{\Gamma (\gamma +2)}\\ +z^{2}\frac{t^{\gamma }}{\Gamma (\gamma +1)}-z^{2}\frac{t^{\gamma +1}}{\Gamma (\gamma +2)}. \end{array}$$

*The subsequent terms are*

(43) $$\begin{array}{c} \upsilon_{2}\left(x,y,z,t\right)=x^{2}\frac{t^{2\gamma }}{\Gamma (2\gamma +1)}+x^{2}\frac{t^{2\gamma +1}}{\Gamma (2\gamma +2)}+y^{2}\frac{t^{2\gamma }}{\Gamma (2\gamma +1)}+y^{2}\frac{t^{2\gamma +1}}{\Gamma (2\gamma +2)}\\ +z^{2}\frac{t^{2\gamma }}{\Gamma (2\gamma +1)}-z^{2}\frac{t^{2\gamma +1}}{\Gamma (2\gamma +2)},\\ \upsilon_{3}\left(x,y,z,t\right)=x^{2}\frac{t^{3\gamma }}{\Gamma (3\gamma +1)}+x^{2}\frac{t^{3\gamma +1}}{\Gamma (3\gamma +2)}+y^{2}\frac{t^{3\gamma }}{\Gamma (3\gamma +1)}+y^{2}\frac{t^{3\gamma +1}}{\Gamma (3\gamma +2)}\\ +z^{2}\frac{t^{3\gamma }}{\Gamma (3\gamma +1)}-z^{2}\frac{t^{3\gamma +1}}{\Gamma (3\gamma +2)},\\ .\\ .\\ . \end{array}$$

*The NTDM solution for Example 6 is*

$$\upsilon \left(x,y,z,t\right)=\upsilon_{0}\left(x,y,z,t\right)+\upsilon_{1}\left(x,y,z,t\right)+\upsilon_{2}\left(x,y,z,t\right)+\upsilon_{3}\left(x,y,z,t\right)+\cdots$$

$$\begin{array}{c} \upsilon \left(x,y,z,t\right)=\left(x^{2}+y^{2}-z^{2}\right)t+x^{2}\frac{t^{\gamma }}{\Gamma (\gamma +1)}+x^{2}\frac{t^{\gamma +1}}{\Gamma (\gamma +2)}+y^{2}\frac{t^{\gamma }}{\Gamma (\gamma +1)}+y^{2}\frac{t^{\gamma +1}}{\Gamma (\gamma +2)}\\ +z^{2}\frac{t^{\gamma }}{\Gamma (\gamma +1)}-z^{2}\frac{t^{\gamma +1}}{\Gamma (\gamma +2)}+x^{2}\frac{t^{2\gamma }}{\Gamma (2\gamma +1)}+x^{2}\frac{t^{2\gamma +1}}{\Gamma (2\gamma +2)}+y^{2}\frac{t^{2\gamma }}{\Gamma (2\gamma +1)}+y^{2}\frac{t^{2\gamma +1}}{\Gamma (2\gamma +2)}\\ +z^{2}\frac{t^{2\gamma }}{\Gamma (2\gamma +1)}-z^{2}\frac{t^{2\gamma +1}}{\Gamma (2\gamma +2)}+x^{2}\frac{t^{3\gamma }}{\Gamma (3\gamma +1)}+x^{2}\frac{t^{3\gamma +1}}{\Gamma (3\gamma +2)}+y^{2}\frac{t^{3\gamma }}{\Gamma (3\gamma +1)}+y^{2}\frac{t^{3\gamma +1}}{\Gamma (3\gamma +2)}\\ +z^{2}\frac{t^{3\gamma }}{\Gamma (3\gamma +1)}-z^{2}\frac{t^{3\gamma +1}}{\Gamma (3\gamma +2)}. \end{array}$$

*This result is calculated to get the exact solution in a closed form:*

$$\upsilon \left(x,y,z,t\right)=\left(x^{2}+y^{2}\right)e^{t}+z^{2}e^{-t}-\left(x^{2}+y^{2}+z^{2}\right).$$

**Figure 7:**
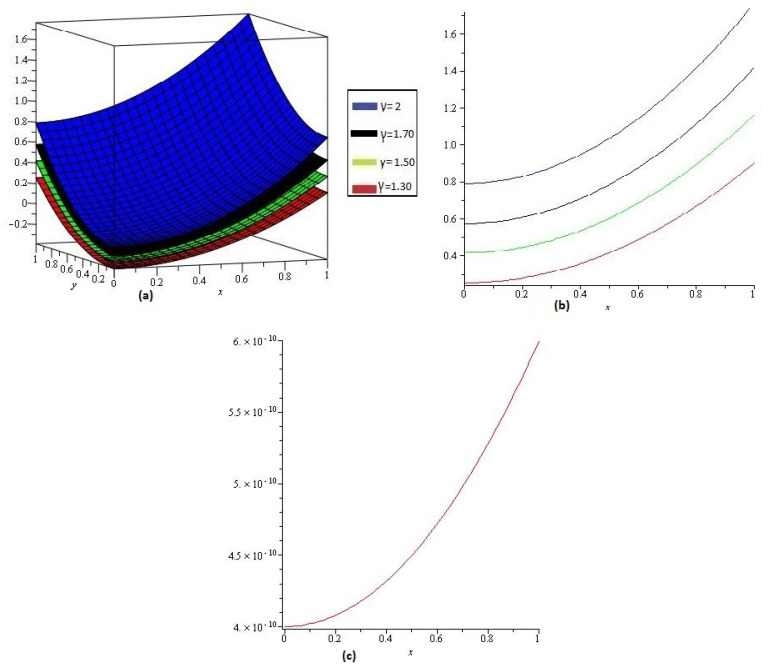
(**a**) Comparison with numerical solution of υ(x,y,z,t) by NTDM of Example 6, for different values of γ and (**b**) for t=1; (**c**) error plot of Example 6 for γ=1.

Figure 7a,b shows the behavior of obtained solution υ(x,y,z,t) by the proposed method for different values of γ=2, 1.80, 1.70, 1.50 and z,t=1; Figure 7c error plot for γ=2 the range of *x* and *y* 0<x,y≤1.

**Example 7.**  *Consider the two-dimensional fractional-order nonlinear wave equation [25]:*

(44) $$\frac{\partial^{\gamma }\upsilon }{\partial t^{\gamma }}=\frac{\partial^{2}}{\partial x\partial y}\left(\upsilon_{xx}\upsilon_{yy}\right)-\frac{\partial^{2}}{\partial x\partial y}\left(xy\upsilon_{x}\upsilon_{y}\right)-\upsilon ,\;0<\gamma \leq 2,$$

*with initial condition*

(45) $$\upsilon \left(x,y,0\right)=e^{xy},\;\upsilon_{t}\left(x,y,0\right)=e^{xy}.$$

*Taking natural transform of Equation (44),*

$$\frac{s^{\gamma }}{u^{\gamma }}N^{+}\left[\upsilon (x,y,t)\right]-\frac{s^{\gamma -1}}{u^{\gamma }}\upsilon (x,y,0)-\frac{s^{\gamma -2}}{u^{\gamma -1}}\upsilon_{t}\left(x,y,0\right)=N^{+}\left[\frac{\partial^{2}}{\partial x\partial y}\left(\upsilon_{xx}\upsilon_{yy}\right)-\frac{\partial^{2}}{\partial x\partial y}\left(xy\upsilon_{x}\upsilon_{y}\right)-\upsilon \right].$$

*Applying inverse natural transform,*

$$\upsilon (x,y,t)=N^{-}\left[\frac{\upsilon (x,y,0)}{s}+\frac{u\upsilon_{t}\left(x,y,0\right)}{s^{2}}+\frac{u^{\gamma }}{s^{\gamma }}N^{+}\left[\frac{\partial^{2}}{\partial x\partial y}\left(\upsilon_{xx}\upsilon_{yy}\right)-\frac{\partial^{2}}{\partial x\partial y}\left(xy\upsilon_{x}\upsilon_{y}\right)-\upsilon \right]\right].$$

*Using the ADM procedure, we get*

$$\upsilon_{0}\left(x,y,t\right)=N^{-}\left[\frac{\upsilon (x,y,0)}{s}+\frac{u\upsilon_{t}\left(x,y,0\right)}{s^{2}}\right]=N^{-}\left[\frac{e^{xy}}{s}+\frac{ue^{xy}}{s^{2}}\right],$$

(46) $$\upsilon_{0}\left(x,y,t\right)=e^{xy}+e^{xy}t,$$

$$\sum_{j=0}^{\infty }\upsilon_{j+1}\left(x,y,t\right)=N^{-}\left[\frac{u^{\gamma }}{s^{\gamma }}N^{+}\left[\frac{\partial^{2}}{\partial x\partial y}\left(\sum_{j=0}^{\infty }A_{j}\left(\upsilon_{xx}\upsilon_{yy}\right)\right)-\frac{\partial^{2}}{\partial x\partial y}\left(xy\sum_{j=0}^{\infty }B_{j}\left(\upsilon_{x}\upsilon_{y}\right)\right)-\sum_{j=0}^{\infty }\upsilon_{j}\right]\right],$$

*where A and B are nonlinear terms. The few nonlinear terms are as follows:*

$$\begin{array}{c} A_{0}\left(\upsilon_{xx}\upsilon_{yy}\right)=\upsilon_{xx}\left(0\right)\upsilon_{yy}\left(0\right),\\ A_{1}\left(\upsilon_{xx}\upsilon_{yy}\right)=\upsilon_{xx}\left(0\right)\upsilon_{yy}\left(1\right)+\upsilon_{xx}\left(1\right)\upsilon_{yy}\left(0\right),\\ A_{2}\left(\upsilon_{xx}\upsilon_{yy}\right)=\upsilon_{xx}\left(0\right)\upsilon_{yy}\left(2\right)+\upsilon_{xx}\left(1\right)\upsilon_{yy}\left(1\right)++\upsilon_{xx}\left(2\right)\upsilon_{yy}\left(0\right), \end{array}$$

*and so on*

$$\begin{array}{c} B_{0}\left(\upsilon_{x}\upsilon_{y}\right)=\upsilon_{x}\left(0\right)\upsilon_{y}\left(0\right),\\ B_{1}\left(\upsilon_{x}\upsilon_{y}\right)=\upsilon_{x}\left(0\right)\upsilon_{y}\left(1\right)+\upsilon_{x}\left(1\right)\upsilon_{y}\left(0\right),\\ B_{2}\left(\upsilon_{x}\upsilon_{y}\right)=\upsilon_{x}\left(0\right)\upsilon_{y}\left(2\right)+\upsilon_{x}\left(1\right)\upsilon_{y}\left(1\right)++\upsilon_{x}\left(2\right)\upsilon_{y}\left(0\right), \end{array}$$

*for j=0*

(47) $$\begin{array}{c} \upsilon_{1}\left(x,y,t\right)=N^{-}\left[\frac{u^{\gamma }}{s^{\gamma }}N^{+}\left[\frac{\partial^{2}}{\partial x\partial y}\left(\frac{\partial^{2}\upsilon_{0}}{\partial x^{2}}\frac{\partial^{2}\upsilon_{0}}{\partial y^{2}}\right)-\frac{\partial^{2}}{\partial x\partial y}\left(xy\frac{\partial \upsilon_{0}}{\partial x}\frac{\partial \upsilon_{0}}{\partial y}\right)-\upsilon_{0}\right]\right],\\ \upsilon_{1}\left(x,y,t\right)=N^{-}\left[-\frac{u^{\gamma }}{s^{\gamma +1}}-\frac{u^{\gamma }}{s^{\gamma +2}}\right]e^{xy}=-e^{xy}\frac{t^{\gamma }}{\Gamma (\gamma +1)}-e^{xy}\frac{t^{\gamma +1}}{\Gamma (\gamma +2)}. \end{array}$$

*The subsequent terms are*

(48) $$\begin{array}{c} \upsilon_{2}\left(x,y,t\right)=N^{-}\left[\frac{u^{\gamma }}{s^{\gamma }}N^{+}\left[\frac{\partial^{2}}{\partial x\partial y}\left(\frac{\partial^{2}\upsilon_{1}}{\partial x^{2}}\frac{\partial^{2}\upsilon_{0}}{\partial y^{2}}+\frac{\partial^{2}\upsilon_{0}}{\partial x^{2}}\frac{\partial^{2}\upsilon_{1}}{\partial y^{2}}\right)-\frac{\partial^{2}}{\partial x\partial y}\left(xy\frac{\partial \upsilon_{0}}{\partial x}\frac{\partial \upsilon_{1}}{\partial y}+xy\frac{\partial \upsilon_{1}}{\partial x}\frac{\partial \upsilon_{0}}{\partial y}\right)-\upsilon_{0}\right]\right],\\ \upsilon_{2}\left(x,y,t\right)=e^{xy}\frac{t^{2\gamma }}{\Gamma (2\gamma +1)}+e^{xy}\frac{t^{2\gamma +1}}{\Gamma (2\gamma +2)},\\ \upsilon_{3}\left(x,y,t\right)=N^{-}[\frac{u^{\gamma }}{s^{\gamma }}N^{+}[\frac{\partial^{2}}{\partial x\partial y}\left(\frac{\partial^{2}\upsilon_{0}}{\partial x^{2}}\frac{\partial^{2}\upsilon_{2}}{\partial y^{2}}+\frac{\partial^{2}\upsilon_{1}}{\partial x^{2}}\frac{\partial^{2}\upsilon_{1}}{\partial y^{2}}+\frac{\partial^{2}\upsilon_{2}}{\partial x^{2}}\frac{\partial^{2}\upsilon_{0}}{\partial y^{2}}\right)-\frac{\partial^{2}}{\partial x\partial y}(xy\frac{\partial \upsilon_{0}}{\partial x}\frac{\partial \upsilon_{2}}{\partial y}+xy\frac{\partial \upsilon_{1}}{\partial x}\frac{\partial \upsilon_{1}}{\partial y}\\ +xy\frac{\partial \upsilon_{2}}{\partial x}\frac{\partial \upsilon_{0}}{\partial y})-\upsilon_{0}]]\\ \upsilon_{3}\left(x,y,t\right)=-e^{xy}\frac{t^{3\gamma }}{\Gamma (3\gamma +1)}-e^{xy}\frac{t^{3\gamma +1}}{\Gamma (3\gamma +2)}. \end{array}$$

*The NTDM solution for Example 7 is*

$$\upsilon \left(x,y,t\right)=\upsilon_{0}\left(x,y,t\right)+\upsilon_{1}\left(x,y,t\right)+\upsilon_{2}\left(x,y,t\right)+\upsilon_{3}\left(x,y,t\right)+\upsilon_{4}\left(x,y,t\right)\cdots$$

$$\begin{array}{c} \upsilon \left(x,y,t\right)=e^{xy}+e^{xy}t-e^{xy}\frac{t^{\gamma }}{\Gamma (\gamma +1)}-e^{xy}\frac{t^{\gamma +1}}{\Gamma (\gamma +2)}+e^{xy}\frac{t^{2\gamma }}{\Gamma (2\gamma +1)}+e^{xy}\frac{t^{2\gamma +1}}{\Gamma (2\gamma +2)}\\ -e^{xy}\frac{t^{3\gamma }}{\Gamma (3\gamma +1)}-e^{xy}\frac{t^{3\gamma +1}}{\Gamma (3\gamma +2)}. \end{array}$$

*This result is calculated to get the exact solution in a closed form:*

$$\upsilon \left(x,y,t\right)=e^{xy}\left(\sin t+\cos t\right).$$

**Figure 8:**
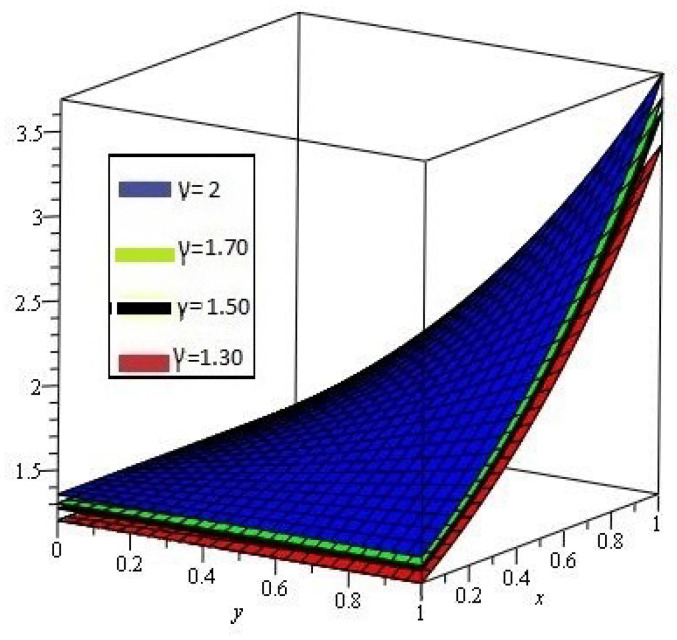
Comparison with numerical solution of υ(x,y,t) by NTDM of Example 7, for different values of γ for t=1.

*Figure 8 shows the behavior of obtained solution υ(x,y,t) by the proposed method for different values of γ=2, 1.80, 1.70, 1.50 and t=1.*

## 5. Conclusions

In this paper, the analytical solutions of fractional-order heat and wave equations are determined, using NTDM. The NTDM solutions are obtained at fractional and integer orders for all problems. The results revealed the highest agreement with the exact solutions for the problems. The NTDM solutions for some numerical examples have shown the validity of the proposed method. It is also investigated that the fractional order solutions are convergent to the exact solution for the problems as fractional order approaches to integer order. The implementation of NTDM to illustrative examples have also confirmed that the fractional order mathematical model can be the best representation of any experimental data as compared to integer order model. In the future, NTDM can be used to find the analytical solution of other nonlinear FPDEs, which are frequently used in science and engineering. NTDM solutions for fractional order problems will prove better understanding of the real world problems represented by FPDEs.
